# Structural Insights Into the Function of *Leishmania major* Adenylosuccinate Lyase

**DOI:** 10.1002/prot.70150

**Published:** 2026-06-08

**Authors:** Ivan R. e Silva, Monique Mantovani, Maria E. S. D. Lino, Aline O. Albuquerque, João H. M. da Silva, Ronaldo A. P. Nagem, Adriana L. Rojas, Geraldo R. Sartori, Eduardo Horjales, David Campbell, Otavio H. Thiemann

**Affiliations:** ^1^ Institute of Physics of São Carlos, University of São Paulo São Carlos, São Paulo Brazil; ^2^ Laboratory of Structural and Functional Biology Applied to Biopharmaceuticals Fundação Oswaldo Cruz, Fiocruz Ceará Eusébio Brazil; ^3^ Pasteur‐Fiocruz Center on Immunology and Immunotherapy Fundação Oswaldo Cruz, Fiocruz Ceará Eusébio Brazil; ^4^ Department of Biochemistry and Immunology Federal University of Minas Gerais Belo Horizonte Minas Gerais Brazil; ^5^ Microbiology, Immunology & Molecular Genetics University of California, Los Angeles Los Angeles CA USA

**Keywords:** Adenylosuccinate lyase, ASL, *Leishmania major*, purine synthesis pathway, *Trypanosoma brucei*

## Abstract

One of several intriguing aspects of kinetoplastid biochemistry is the complete dependence on host purines and purine recycling due to the lack of a *de novo* purine biosynthesis pathway. Adenylosuccinate lyase (ASL, EC 4.3.2.2) is a key enzyme in the purine synthesis pathway responsible for the conversion of adenylosuccinate into adenosine monophosphate (AMP), representing a potential target for an effective drug design against leishmaniasis. Here, we report the in vitro kinetics studies and the crystal structure of the *Leishmania major* Friedlin adenylosuccinate lyase (*Lm*ASL). Furthermore, we characterize allosteric communication networks within the protein. We propose a phenylpiperazine derivative, itraconazole, as a promising candidate for selective interaction with the *Lm*ASL substrate‐binding site by molecular docking and molecular dynamics simulations. Finally, we expand the current understanding on trypanosomatid ASL by demonstrating its requirement for the normal growth of *Trypanosoma brucei* procyclic form. Our data will substantiate future studies aimed at developing an effective and specific treatment against leishmaniasis.

## 1 Introduction

Kinetoplastid parasites such as *Leishmania* sp., *Trypanosoma brucei*, and *Trypanosoma cruzi* are responsible for thousands of productive life years lost worldwide due to serious diseases in a wide variety of hosts [1]. *Leishmania* species, for instance, are the causative agents for human visceral and skin leishmaniasis with high incidence in the tropical and subtropical regions. The treatment and control of such parasitic diseases are severely compromised by the dearth of effective and selective antiparasitic therapies [2, 3]. Such a development is dependent on the knowledge of the biochemical pathways and the enzymes involved.

**FIGURE 1:**
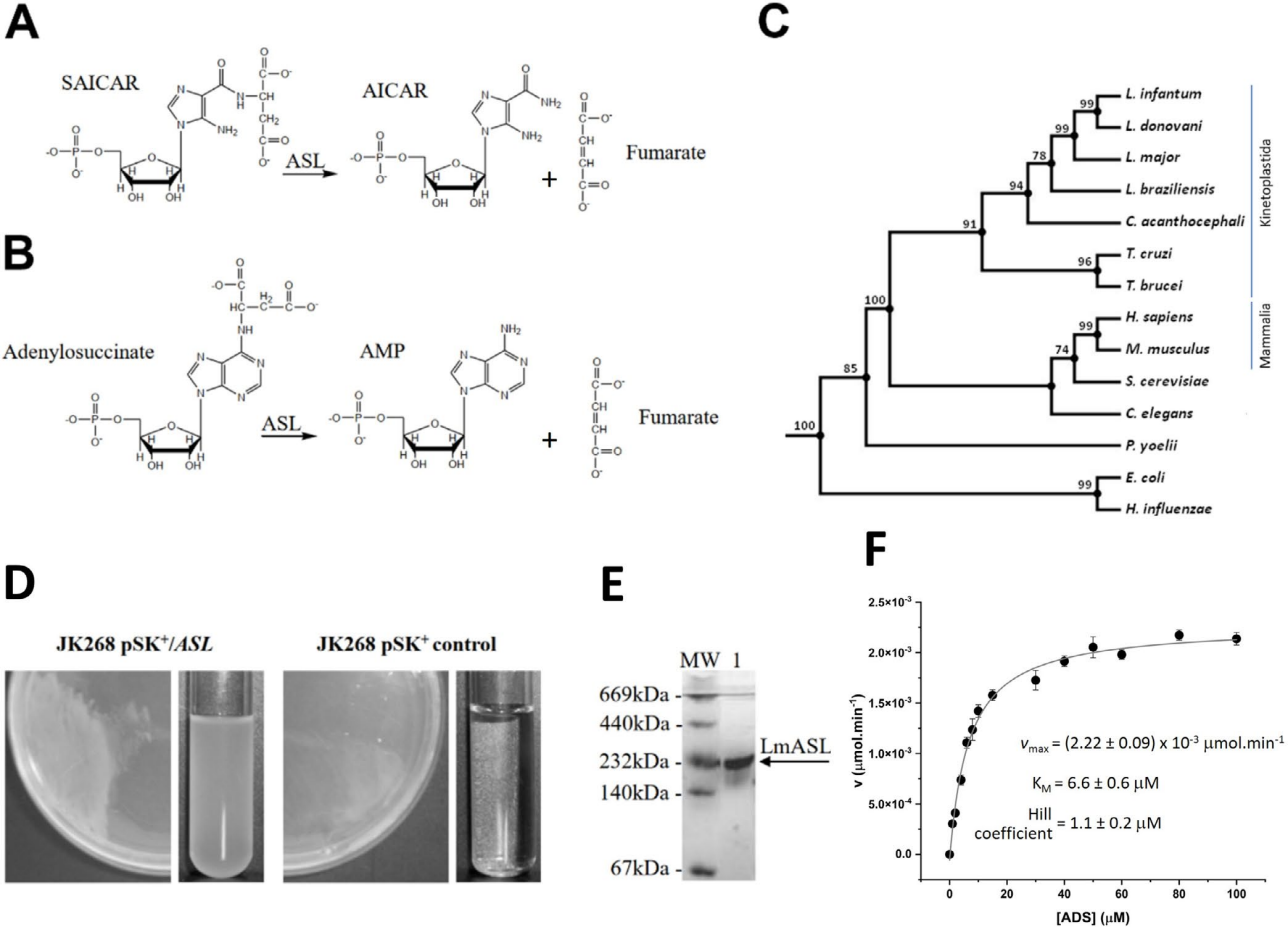
*Lm*ASL function. (A, B) Reactions typically catalyzed by adenylosuccinate lyase in the de novo purine synthesis pathways. (C) ASL protein phylogenetic tree showing a Maximum Likelihood analysis of homologous ASL sequences. Bootstrap confidence values are indicated for 1000 iterations. (D) *Lm*ASL functional complementation in *asl‐* *Escherichia coli* JK268. Growing bacteria transformed with the recombinant plasmid (pSK+/*asl*) in solid (left) and liquid (right) minimal media‐M9. JK268 cells transformed with the empty vector (pSK+ 612 without the insert) were also inoculated as a negative control. (E) Native gel electrophoresis analysis showing a recombinant tetrameric protein. (F) Michaelis–Menten plot of initial reaction velocities (v) of product formation using ADS as substrate.

Interestingly, these human parasites lack a *de novo* nucleotide biosynthesis pathway and largely rely on the preformed bases and nucleosides from their hosts [4]. In humans, adenylosuccinate lyase (*Hs*ASL, EC 4.3.2.2) is a bifunctional enzyme that catalyzes two non‐sequential reactions of purine biosynthesis [5]. *Hs*ASL catalyzes the conversion of N‐succinylo‐5‐aminoimidazole‐4‐carboxamide ribonucleotide (SAICAR) into 5‐aminoimidazole‐4‐carboxamide ribonucleotide (AICAR) and fumarate (Figure 1A) in the *de novo* synthesis of inosine monophosphate (IMP). *Hs*ASL is also involved in the conversion of succinyladenosine monophosphate (SAMP) to adenosine monophosphate (AMP) and fumarate in the purine synthesis pathway (Figure 1B) [5, 6, 7, 8].

In trypanosomatids, the purine synthesis pathway is distributed between the cytosol and the glycosome [9] and ASL is the last cytosolic enzyme involved in AMP synthesis. As expected for such an important enzyme, ASL knockout impairs *L. donovani* growth and results in a lower capacity to infect mice [10] thus representing a potential target for the development of effective drugs against leishmaniasis. Whether ASL is essential for other trypanosomatid parasites has remained elusive.

*In silico* studies have recently demonstrated the effectiveness of computational approaches for identifying potential enzyme inhibitors across oncogenic, metabolic, and parasitic contexts. For example, curcumin and the 5‐AZA‐CdR (5‐Aza‐2′‐desoxicitidina) compound exhibited significant interaction with human DNA metiltransferase (DNMT1) in molecular docking and MD simulations, corroborated by in vitro activity assays in lung adenocarcinoma cells [11]. Similarly, screening of 25 000 compounds against human cyclin‐dependent kinase 4 (CDK4) in retinoblastoma identified promising inhibitors based on molecular docking scores, binding modes, and MD analyses, highlighting the utility of computational methods for initial enzyme inhibitor candidate selection [12]. Computational screening has also proven cost‐ and time‐efficient, as illustrated in studies targeting human fatty acid transport protein 2 (FATP2), an enzyme involved in hyperglycemia and diabetic nephropathy [13]. In parasitic research, virtual screening, MD simulations, and MM‐GBSA calculations recently identified two natural‐product‐derived compounds binding stably to N‐acetylglucosamine‐phosphatidylinositol de‐N‐acetylase (NAGP‐deacetylase) in *Leishmania donovani*, a key enzyme in GPI biosynthesis, supporting their potential as selective antiparasitic scaffolds. Together, these findings demonstrate that molecular docking and molecular dynamics simulations provide a robust and versatile framework for the early identification of enzyme inhibitors across diverse disease models.

In this paper, we present the crystal structure and in vitro kinetics of *L. major* adenylosuccinate lyase (*Lm*ASL) and compare it with available homologs to determine the amino acid residues involved in catalysis and oligomerization. We further analyze the allosteric communication networks within *Lm*ASL that are responsible for its function. Kinetoplastid ASL proteins are evolutionarily distant from the human host ASL, reinforcing the potential for the development of an effective and specific inhibitor of the parasite enzyme. In fact, a phenylpiperazine derivative, itraconazole, was identified as a promising selective binder of the protein's substrate‐binding site in molecular docking and molecular dynamics simulations. Furthermore, we show that ASL is also essential for the viability of procyclic *T. brucei*, opening a new avenue for the development of novel drugs specific for the parasite ASL.

## 2 Materials and Methods

### 2.1 Cloning

Specific oligonucleotides (*Lm*ASL1–5′‐ATGTCACTGCCTTCGGCACAAGAAG‐3′, *Lm*ASL2–5′‐TCAGGGACGCGCCGTGTAGC‐3′, *Tb*ASL1–5′‐ACTCGGATCCATGGAGAAGGGTTCACCATCCGAC TTAAAC‐3′, *Tb*ASL2–5′‐CTACTCGAGTTTAGCACTGAAGCGAGGAACGTACCCTAC‐3′) were synthesized based on the ASL open reading frames identified from the genomic sequence data of *L. major* and *Trypanosoma brucei* TREU927, NCBI [14] accession numbers FR796400 and XM_822015, respectively. The ASL sequences were amplified by PCR in a PTC‐100 thermocycler (MJ 110 Research Inc.) with 0.2 U of Taq DNA polymerase (Invitrogen) according to the manufacturer's conditions and cloned into the pCR‐TOPO/T7‐NT expression vector (Invitrogen) for the *L. major* ASL and into the pGEM‐T vector (Promega) for the *T. brucei* ASL.

### 2.2 Phylogenetic Reconstruction

The amino acid sequence of *L. major* ASL (NCBI accession number FR796400) was used to search the NCBI protein database [14] for homologous ASLs. The ASL of *L. infantum* (XP_001462882.1), *L. donovani* (*Ld*ASL, XP_003858107.1), *L. braziliensis* (XP_001561734.1), *Crithidia acanthocephali* (AGT02638.1), *T. cruzi* (Q4CXJ7), *T. brucei* *gambiense* (XP_011776602.1), *Homo sapiens* (P30566.2), *Escherichia coli* (P0AB89.1), *Saccharomyces cerevisiae* (Q05911.1), *Caenorhabditis elegans* (Q21774.1), *Mus musculus* (P54822), *Haemophilus influenzae* (P44797.1), and *Plasmodium yoelii* (Q7RC16). The protein sequences were aligned with Clustal Omega [15] with default parameters. The aligned sequences were visually examined. The best fit evolutionary model was determined by ModelTest [16]. The Maximum Likelihood tree was calculated by the stochastic algorithm of IQ‐TREE [17]. Bootstrap confidence values were calculated with 1000 iterations [18] and a consensus tree was obtained.

### 2.3 Recombinant LmASL Expression and Purification

The recombinant expression plasmid (p*Lm*ASL) was transformed into *E. coli* BL21 (DE3) pLysS competent cells (Invitrogen). Cells from a single colony were grown in 5 mL of LB media containing 100 μg.mL^−1^ ampicillin and 34 μg.mL^−1^ chloramphenicol, at 37°C, 250 rpm agitation for 15 h. This culture was used to inoculate 1 L of 2xYT media, containing 0.2% glucose and the same antibiotics as described above. The cells were grown at 37°C and 250 rpm until the culture reached an OD_600_ of approximately 0.6, at which point recombinant *L. major* ASL (r*Lm*ASL) expression was induced by the addition of 0.1 mM IPTG at 25°C for 5 h. The induced cells were centrifuged at 5000 ***g*** for 15 min and stored at −80°C. Frozen cell pellets from 1 L of culture were suspended in 20 mL of buffer A (50 mM potassium phosphate, pH 7.8, 400 mM NaCl, 100 mM KCl, 10% glycerol, 10 mM imidazole, 150 U of DNase) and lysed by 6–8 cycles of freeze‐thawing. The lysate was centrifuged at 20 000 ***g*** for 30 min and the supernatant transferred to a fresh tube. Expression efficiency was analyzed in a 15% SDS‐PAGE and visualized by Coomassie blue staining. The protein supernatant, of approximately 20 mL, was loaded into a 5 mL NTA immobilized metal‐chelate affinity chromatography column (Ni‐NTA Superflow resin—Qiagen) pre‐equilibrated with 4 column volumes (CV) of buffer A. The column was washed with 4 CV of buffer A containing 25 mM imidazole. Recombinant protein was eluted from the column with 2 CV of buffer A containing 500 mM imidazole and dialyzed against multiple changes of buffer B (50 mM potassium phosphate, pH 7.8, 200 mM NaCl and 1 mM DTT). r*Lm*ASL was concentrated by ultrafiltration using a centriprep 30 (Millipore) to a final volume of 2 mL and applied to a pre‐equilibrated HiLoad16/600 Superdex 200 (Cytiva) size exclusion chromatography column at a flow rate of 0.5 mL/min. Fractions were collected across the peak and analyzed by 15% SDS‐PAGE stained by Coomassie Blue. Protein concentrations were determined by the Bradford's method, using the Bio‐Rad protein 230 assay dye reagent according to the manufacturer's instructions. Isoelectric focusing (PhastSystem—GE Healthcare) was used to determine the experimental pI of r*Lm*ASL. Protein aliquots of 4.0 μL at 1.0 mg/mL, in buffer B were applied to Phast Gel IEF 3–9 and stained by Coomassie Blue. The N‐terminal sequence containing the pCR‐TOPO/T7‐NT (Invitrogen) encoded histidine sequence was cleaved by proteolytic digestion with enterokinase (Invitrogen) at a proportion of 40 μg *Lm*ASL to 8 U enterokinase, in a final volume of 60 μL, for 24 h at 18°C. The product of the reaction was separated in a 15% SDS‐PAGE and the protein bands were transferred to a PVDF (Bio‐Rad) membrane. The membrane was stained with 0.5% Ponceau red and the immobilized bands were submitted to Edman Degradation N‐terminal sequencing, using a Procise Model 491‐PE (Applied Biosystems), at the Protein Chemistry Centre, School of Medicine/USP, at Ribeirão Preto/SP (Brazil).

### 2.4 LmASL Crystallization, Data Collection and Structure Determination

Crystals of r*Lm*ASL were obtained by the vapor diffusion method at 18°C using 3 μL of protein solution at 10 mg.mL^−1^ (in a buffer containing 5 mM K_3_PO_4_, 5 mM NaCl, 100 μM ADS) and 3 μL of reservoir solution (1.6 M ammonium sulfate, 0.1 M MES pH 6.5, 10% dioxane). Crystals were mounted in the mother liquor, to which ethylene glycol was added to 20%, and flash‐cooled in liquid nitrogen. In‐house data collection was performed in a Rigaku UltraX 18 rotating anode X‐ray generator equipped with a MAR345 dtb image plate at 1.542 Å. The resulting dataset was integrated using iMosflm [19] and scaled using AIMLESS [20]. The structure was solved by molecular replacement with PHASER [21] using the crystal structure of *Tb*ASL (PDB 4EFC [22]) with removed loops and a poly‐L‐alanine model as a search model. Cycles of refinement in REFMAC [23], PHENIX.REFINE [24], including TLS refinement (6 groups), and manual building in COOT [25] allowed the complete building of the crystal structure. The crystal structure of *L. major* ASL was deposited in the PDB [26] under the accession code 9BPL. A homotetramer model was generated using PDBePISA [27]. Structural analysis was proceeded in the PyMOL Molecular Graphics System (Version 3.0, Schrödinger LLC) and APBS [28].

### 2.5 Mapping Allosteric Communication Networks Within LmASL

We submitted the atomic model of the *Lm*ASL tetramer generated by PISA [27] to the analysis of allosteric communication networks in the Ohm server [29], which uses percolation theory to identify amino acid residue connections throughout the protein. The frequency with which each residue in a network is affected by a perturbation is calculated as the allosteric coupling intensity (ACI) [29]. The allosteric paths analysis was continued by selecting the amino acid residue His196 from chain 1 as the active site of interest and independently setting Arg40 from either chain 2, 3, or 4 as putative allosteric sites.

### 2.6 Kinetics Analysis of LmASL

The enzyme assay method was modified from the previously developed assay [30]. Activity of the r*Lm*ASL was assayed in biological replicates (3X) in a 500 μL reaction buffer C (50 mM Tris–HCl, pH 8.5), at 25°C by monitoring the decrease in absorbance at 282 nm for 1 min using a UV visible spectrophotometer (UV‐1650 PC, Schimadzu). The extinction coefficient difference between adenylosuccinate (substrate) and AMP (product) is 10 000 M^−1^ cm^−1^ [30] and with an r*Lm*ASL concentration of 1.6 nM. The concentration of adenylosuccinate varied between 1.0 and 100 μM. SAICAR substrate was used at a concentration of 70 μM in the same buffer conditions as described. The Hill plot was obtained to determine the kinetic parameters values (*K*_M_ and *V*_max_), the kinetic efficiency (*k*_cat_/*K*_M_), and the Hill coefficient for adenylosuccinate.

### 2.7 ASL Functional Complementation in *E. coli* JK268

The *asl* sequence for the functional complementation experiments was amplified by PCR using a standard reaction mixture previously described and 0.2 M of each primer ASL‐*Lm*‐*Xba*I (5′‐GCTCTAGAGATGTCACTGCCTTCGGCAC‐3′) and ASL‐*Lm*‐*Hind*III (5′‐AGTAAGCTTCTATCAGGGACGCGCCGTGTAG‐3′). The amplified DNA was digested with *Xba*I and *Hind*III restriction endonucleases for 4 h at 37°C, gel‐purified by Perfectprep Gel Cleanup Kit (Eppendorf) and cloned into the *Xba*I and *Hind*III digested pBluescript SK+ plasmid (Stratagene). The ligation product was transformed into *E. coli* DH5α competent cells and recombinant clones were selected. The recombinant plasmid pSK/asl and pBluescript SK+ plasmid were used to transform the ASL deficient *E. coli* JK268 (replacement to JK266, dadR1, trpE61, trpA62, tna5, purB58) [31]. *E. coli* JK268 were grown in minimal media M9 (Sigma) containing 100 μg.mL^−1^ ampicilin and supplemented with 0.5% glucose, 0.1% acid hydrolyzed casein, 0.2 μg.mL^−1^ thiamin and 50 μg.mL^−1^ tryptophan [32].

### 2.8 Cell Culture

The *L. major* Friedlin (MHOM/IL/81/Friedlin) cells were grown at 26°C in M199 media (Invitrogen) supplemented with 10% heat inactivated fetal bovine serum (FBS, Invitrogen). Procyclic *T. brucei* 29‐13 cells were grown at 28°C in the Cunningham's medium supplemented with 10% heat inactivated FBS, G418 98 (15 μg.mL^−1^) and hygromycin B (50 μg.mL^−1^).

### 2.9 RNA Interference (RNAi) Knockdown of *T. brucei* ASL

To investigate the effect of the lack of ASL on the parasite's metabolism we used the inducible RNA interference system available for *T. brucei* cells. A 392 bp *Xho*I and *Hind*III digested *T. brucei* *asl* fragment (nucleotides 1024 to 1416) was cloned into the pZJM RNAi vector [33] generating the plasmid pZJM/*Tb*ASL. Transfection of procyclic *T. brucei* 29‐13 cells with *Not*I linearized pZJM/*Tb*ASL DNA was performed as follows: electroporation was carried out by two 25 μF and 1.5 kV consecutive pulses in a 0.4 cm gap cuvette in a Bio‐Rad 185 Gene Pulser II. The electroporated cells were transferred to a 5 mL culture bottle in Cunningham's medium. The transfectants were selected by the addition of 2.5 μg.mL^−1^ phleomycin, and single cells were cloned by limiting dilution. Induction of RNAi expression was done by the addition of 3.0 μg.mL^−1^ tetracycline and the cell numbers of RNAi induced and RNAi uninduced cultures were monitored in a hemocytometer. Total RNA was extracted with the TRIzol reagent (Invitrogen) from RNAi induced (I) and uninduced (Ui) cells at 24, 48, 72 and 100 h after induction. Each RNA (0.5 g) sample was submitted to RT‐PCR using the ONE STEP RT‐PCR (Qiagen) kit according to the manufacturer's instructions, with the oligonucleotides RT‐TbASL (5′‐TCGCCGTATTGTTTATGTCCTGTGATGTCA‐3′), antisense to *T. brucei* ASL mRNA sequence and the SLRNA specific oligonucleotides, to detect the presence of the *asl* transcript. As a control, RT‐PCR was performed to amplify a 244 nt long sequence of the GAPDH (glyceraldehyde 3 phosphate dehydrogenase) mRNA, using GAPDH specific oligonucleotides *Tb*GAPDH‐1 (5′‐CCTCGGGAATGAGATTGATGTCGTTGCTGT‐3′) and *Tb*GAPDH‐2 (5′‐CACATACTCCACACCAAGCTTTCCC‐3′). The PCR products were fractionated in a 2% agarose‐TAE gel. The resulting DNA bands were cloned into the pGEM T vector (Promega) and sequenced to confirm the identity of the amplified DNA fragment by alignment with the genomic sequence flanking the ASL or the control GAPDH gene.

### 2.10 Molecular Docking

Molecular docking experiments were conducted systematically, incorporating protein preparation, binding site prediction, and molecular docking for *Lm*ASL (PDB 9BPL, this paper), *Ld*ASL (PDB 4MX2 [8]), *Tb*ASL (PDB 4EFC [22]), and *Hs*ASL (PDB 4FFX [5]). PDB PISA [27] was used to generate, when necessary, ASL homotetrameric models, enabling a meaningful comparative analysis of docking results across orthologous proteins. The integrity of the active site was preserved in all structures by retaining critical water molecules and cofactors observed in their respective crystal structures, ensuring protein stability and functional relevance in the docking simulations.

First, the target proteins were protonated at pH 7.4 using the PDB2PQR server [34]. Binding site prediction was carried out using Discovery Studio and the CB‐Dock server [35], with the goal of identifying potential binding sites within each protein. A library of 400 ligands from the Pandemic Response Box (PRB) provided by Medicines for Malaria Venture [36] was employed for docking against *Lm*ASL and *Hs*ASL using the GOLD software [37] with default parameters (radius of 10 Å, ChemPLP, and allowing early termination).

The best‐performing ligand for *Lm*ASL and the worst‐performing ligand for *Hs*ASL were selected for further docking to *Lm*ASL, *Hs*ASL, *Tb*ASL, and *Ld*ASL, using the CBDock server [35]. To further evaluate the interactions of the best‐selected compound with *Lm*ASL and its homologous proteins, we performed docking using two approaches: one using the original input file of the ligand (flexible pose) and another using the best‐ranked pose obtained from the initial docking (fixed pose). The performance was evaluated based on the best coordinates of the target and a free docking scenario using the original file. Rescoring was performed using the GOLD scoring function [37], employing default parameters to ensure consistent evaluation across all docking experiments. A structural analysis of the docking results was performed using the PyMOL Molecular Graphics System (Version 3.0, Schrödinger LLC) and the CB‐Dock [35] interface for visualization and interpretation.

### 2.11 Molecular Dynamics Simulations

Molecular dynamics simulations were performed using the CUDA version of AMBER24 [38] with PMEMD, employing the ff19SB force field [39] for the protein and the gaff2 force field [40] for the ligand. The molecule's formal charge at a physiological pH of 7.4 was predicted using MarvinSketch (v.16.9.26–2016, ChemAxon). Subsequently, the RESP method was used to calculate ligand atomic charges at the B3LYP/6‐31G** level of theory using single‐point energy calculations in the PSI4 quantum chemistry software [41]. Ligand parameters and topology files were generated with the Antechamber package from AmberTools [38]. The preparation of the protein included fixing structural gaps with the ChimeraX v.1.95 [42] loop model tool and renaming the protonatable residues according to their dominant protonation state at pH 7.4 with PDB2PQR [43]. C‐terminal residues were capped with NME (methylamine) groups. The protein‐ligand complex was solvated in a truncated octahedral box of OPC water molecules [44], ensuring a minimum distance of 15 Å between any protein atom and the box boundary. Systems were neutralized and adjusted to a 0.15 M NaCl concentration using the LEaP module of AmberTools [38].

The system underwent a multi‐stage minimization and equilibration protocol. Initially, energy minimization was performed in three stages. The first one applied positional restraints of 500 kcal.mol^−1^.Å^−2^ to the entire solute, including the ligand, minimizing water and ions for 2000 steps. The second one repeated this minimization with restraints on the solute backbone while allowing the ligand to relax. In the final minimization stage, all restraints were removed, and 20 000 minimization steps were carried out. Following minimization, the system was gradually heated from 0 to 310 K over 500 ps under an NVT ensemble with a 1.0 kcal/mol·Å^2^ restraint on all heavy atoms, using Langevin dynamics with a collision frequency of 1.0 ps^−1^.

Equilibration was then performed under an NPT ensemble in multiple stages. First, the system underwent 1 ns of equilibration with a 100 kcal/mol·Å^2^ restraint on the solute, followed by an additional 1 ns with the restraint force constant reduced to 10 kcal.mol^−1^.Å^−2^. Subsequently, three rounds of 1 ns equilibration were carried out with decreasing harmonic restraints on backbone atoms set at 10, 1, and 0.1 kcal.mol^−1^.Å^−2^, respectively. Finally, a 1 ns unrestrained equilibration phase was performed to fully relax the system under constant pressure. Production simulations were conducted in the NPT ensemble for 100 ns in five replicates, saving coordinates every 10 ps. A 10 Å cutoff was used for nonbonded interactions. All bonds involving hydrogen atoms were constrained with the SHAKE algorithm [45], and Hydrogen Mass Repartitioning (HMR) allowed a 4 fs integration time step [46, 47]. Langevin dynamics with a collision frequency of 2 ps^−1^ was used for temperature regulation, and a Monte Carlo barostat was applied for pressure coupling.

Analyses were performed with the cpptraj module [48] of AmberTools. RMSD was calculated with respect to the initial molecular docking pose and to the last frame of each replicate. Distances between the ligand and key active site residues were measured. Principal Component Analysis (PCA) was conducted by first aligning all trajectory frames to the initial minimized structure using Cα atoms. Projections onto the first two principal components (PC1 and PC2) were used to identify dominant motions in the loop comprising amino acid residues Asp311‐Pro330.

## 3 Results

### 3.1 Phylogenetic Analysis of Adenylosuccinate Lyases

We performed an evolutionary analysis of the adenylosuccinate lyase (ASL) homologs using the Maximum Likelihood approach from an alignment of 14 amino acid sequences with 542 sites of which 391 are parsimony informative sites and only 10.3% are constant or ambiguous sites. The best‐fit model calculated by ModelFinder [13] was LG + G4 with a Gamma rate heterogeneity, with 4 categories, and an alpha shape of 1.1201. The obtained consensus phylogenetic tree (Figure 1C) had a total length of 9.3175 after 1000 bootstrap rounds. Interestingly, the Apicomplexa *Plasmodium yoelii* ASL is an early diverging enzyme, probably due in part to the strong nucleotide frequency bias that the genus *Plasmodium* has, leading to a stronger deviation in the primary structure. The nematode ( *Caenorhabditis elegans* ), yeast (*Sccharomyces cerevisiae*) and mammal (Human and mice) ASL sequences clustered within the same clade. The bacterial ASLs represent the more divergent clade. Remarkably, we determined that *L. major* ASL efficiently restored adenylosuccinate lyase function in *asl‐Escherichia coli* JK268 strain grown in minimal media (Figure 1D). The Kinetoplastidae ASLs (*Trypanosoma, Leishmania* and *Crithidia*) clustered in a single monophyletic group with strong statistical support, and distant from the mammalian host ASL. Such a large evolutionary distance between the ASLs of protozoans on the one hand and those of mammals on the other may hold a promise for the development of specific *LmASL* inhibitors.

### 3.2 Crystal Structure of the *L. major* Adenylosuccinate Lyase

**FIGURE 2:**
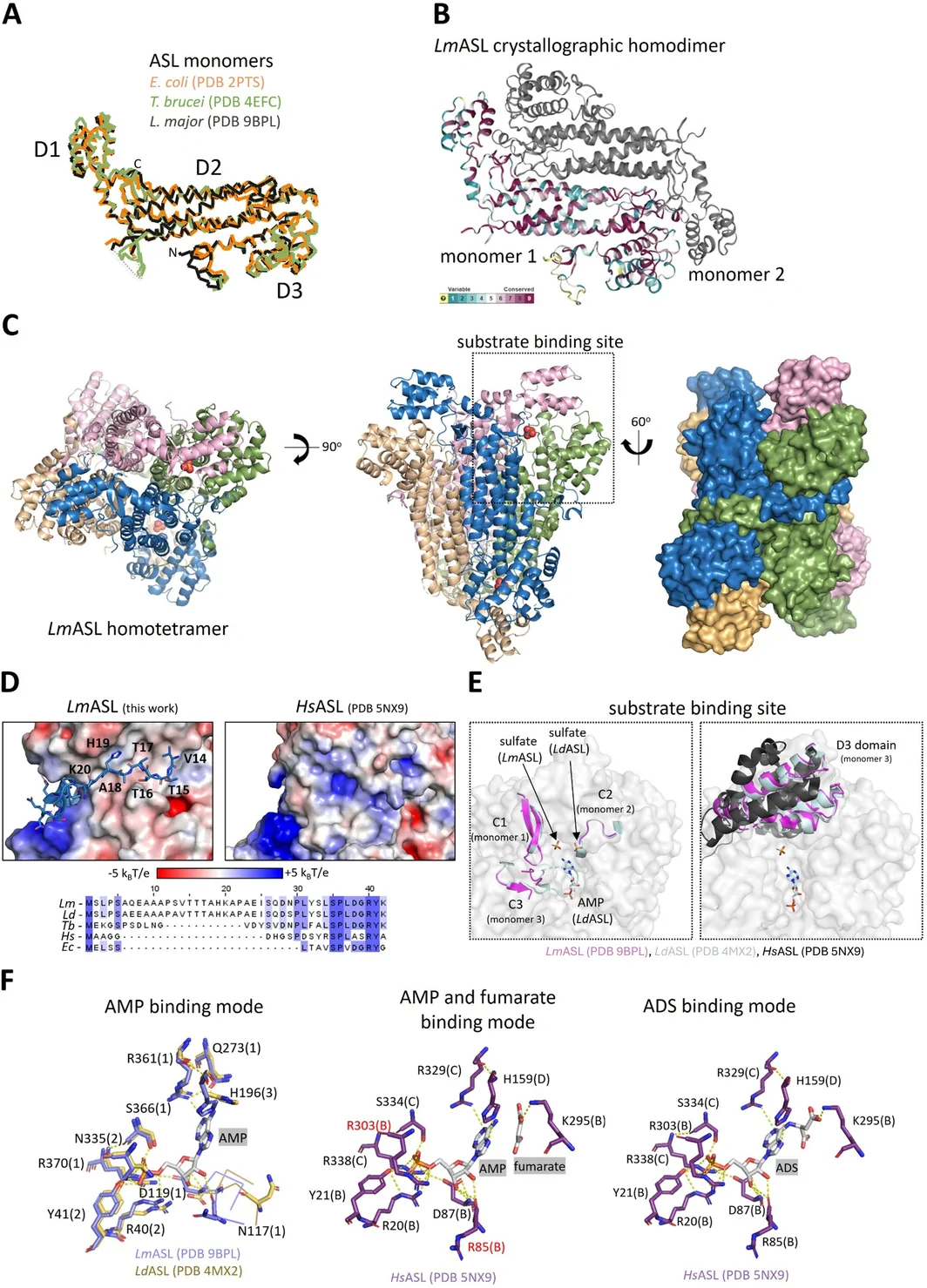
*Lm*ASL crystal structure. (A) Analysis of a monomer of *Lm*ASL superimposed with *Escherichia coli* (PDB 2PTS [49]) and *T. brucei* (PDB 4EFC [22]) ASL monomers. (B) Conservation of the binding interface of the *Lm*ASL crystallographic homodimer, calculated by ConSurf [50]. Only one monomer is colored according to the conservation score, while the other monomer is shown in gray. (C) Cartoon representation of a model of the *Lm*ASL homotetramer generated using the PISA server [24]. The four monomers are colored green, blue, beige and pink. The inset shows domain 3 (*Lm*ASL, cyan) superimposed with PDB 4MX2 (*Ld*ASL, magenta, [8]) in the vicinity of the AMP binding site. (D) Multiple sequence alignment of the N‐terminal region of ASL and comparison of *Lm*ASL and *Hs*ASL (PDB 5NX9 [47]) electrostatic potential surfaces, calculated by APBS [25]. Bound *Lm*ASL N‐terminal region (amino acid residues 14–20) is depicted as sticks. (E) Surface and cartoon representation of the *Lm*ASL active site (PDB 9BPL, this work, magenta), highlighting both the C1‐C3 regions and the D3 domain overlaid to the *Ld*ASL (PDB 4MX2 [8], cyan) and/or *Hs*ASL (PDB 5NX9 [47], black) crystal structures. (F) Comparison of the AMP binding mode of *Ld*ASL (PDB 4MX2 [8], blue) with the amino acids conserved in the same region of *Lm*ASL (PDB 9BPL, this work, yellow). As a reference, AMP/fumarate binding sites and ADS binding site of *Hs*ASL (PDB 5NX9 [47], purple) are also shown. Residues R303 and R85, mutated to N335 and N117, respectively, in *Lm*ASL are shown in red. The PyMOL Molecular Graphics System (Version 3.0—Schrödinger LLC) was employed for protein structure visualization.

To investigate the possible structural differences between *LmASL* and *Hs*ASL that could be used in future structure‐based drug design initiatives, we determined the crystal structure (Figure 2) of *LmASL* at 1.97 Å resolution by molecular replacement using the *T. brucei* ASL structure deposited with PDB (Protein Data Bank) [26] under accession code 4EFC [22] by the Structural Genomics Consortium (SGC) organization as a search model. Data collection and structure refinement statistics are shown in Table 1.

**TABLE 1:** X ray crystallography data collection and refinement statistics.

Data collection^a^	*Lm*ASL
X ray source and detector	RIGAKU ULTRAX 18 MAR scanner 345 mm plate
Space group	I 41 2 2
Wavelength (Å)	1.542
Monomers in the asymmetric unit	2
Unit cell dimensions (Å, ^o^)	*a* = *b* = 130.02, *c* = 316.77 *α* = *β* = *γ* = 90.00
Unique reflections (overall, outer shell)	95 367 (9344)
Resolution (overall/outer shell) (Å)	25.25–1.97 (2.04–1.97)
Completeness (overall/outer shell) (%)	99.32 (99.16)
*R*_merge_ (overall/outer shell)	0.104 (1.373)
*R*_pim_ (overall/outer shell)	0.028 (0.382)
Mean I/*σ* (overall/outer shell)	18.7 (2.2)
Multiplicity (overall/outer shell)	14.2 (13.5)
Wilson B‐factor (Å2)	30.88
*Refinement*	
PDB accession code	9BPL
*Number of atoms (non‐hydrogen)*	
Protein	7089
Solvent	415
Ligands	48
Protein residues	889
Reflections used for R‐free (test set, random)	4732
*R*_work_/*R*_free_	0.211/0.239
Fo, Fc correlation	0.95
*B‐factors (Å* ^*2*^ *)*	
All atoms	42.56
Protein	42.86
Ligands	48.71
Solvent	36.79
*R.M.S.D*	
Bond length (Å)	0.014
Bond angles (^o^)	1.31
*Ramachandran plot (%)*	
Favored regions	96.63
Allowed regions	3.14
Outliers	0.23
Clashscore	4.64

^a^ A single crystal was used for the structure determination.

Although *Hs*ASL and other homologs were reportedly active as homotetramers in solution [5, 6, 49, 51], only two monomers of *LmASL* were present in the crystal asymmetric unit, as also observed for previously published ASL structures under PDB accession codes 1C3U and 1C3C ( *Thermotoga maritima* [52]), 2PTR and 2PTQ ( *E. coli* [53]), 4NLE ( *Mycobacterium smegmatis* [54]), 5EYV ( *Schistosoma mansoni* [55]) and 2HVG (*Plasmodium vivax* [56]). However, a tetrameric functional enzyme structure can be obtained by a two‐fold crystallographic symmetry using PDBePISA [27] (Figure 2C), which might represent the tetrameric organization of the recombinant *Lm*ASL protein in solution, as confirmed by its migration in the native gel electrophoresis (Figure 1E).

In our crystal structure, each *Lm*ASL monomer shows a typical, mostly α‐helical, aspartase/fumarase superfamily fold that consists of an N‐terminal domain (D1, residues 1–100), a central helix bundle (D2, residues 101–366), and a C‐terminal domain (D3, residues 367–474; Figures 2A and S1). Domain D2 is also composed of four β‐strands that are organized as two pairs of antiparallel β‐sheets. A comparison among the *Lm*ASL monomer and ortholog structures deposited in the PDB [26] is shown in Table S1. When the *Lm*ASL crystal structure is compared with *Hs*ASL homologs, the best match (RMSD = 4.4 Å, 26% sequence identity) is observed for PDB 4FFX (unbound *Hs*ASL [5]), demonstrating that indeed the *Lm*ASL structure is more similar to that of protozoan adenylosuccinate lyases than to its human homolog structures.

The two *Lm*ASL monomers observed in the asymmetric unit of the crystal are arranged antiparallel to each other and brought together by a well conserved interface (depicted in violet to green gradient, Figure 2B). The homotetramer model (Figure 2C) generated using the two‐fold crystallographic symmetry presents a buried surface of 36 090 Å^2^. Different from orthologous ASL crystal structures, the N‐terminal extension of the *L. major* enzyme improves the monomer‐monomer interactions in the homotetramer by contacting the adjacent monomer surface (Figure 2C). Amino acid residues from position 14 to 20, absent in *Hs*ASL, interact with a mainly hydrophobic patch on the surface of each monomer, which differs from the human homolog (Figure 2D).

The aspartase/fumarase superfamily fold has been related to ASL due to its ability to catalyze β‐elimination reactions of succinyl‐containing substrates leading to the formation of fumarate. Previously reported adenylosuccinate/AMP + fumarate bound‐ASL structures demonstrated that ASL has four active sites, each one containing amino acid residues from three conserved regions (C1—residues 143–148, C2—residues 190–204 and C3—residues 308–330, Figures 2E and S1) of domain D2 of three contributing monomers [5]. Although *Lm*ASL crystallized in the presence of 100 μM adenylosuccinate in the crystallization condition, our crystal structure instead showed electron density consistent with a sulfate molecule bound at each active site. This is likely due to the high concentration of ammonium sulfate (1.6 M) and 5 mM potassium phosphate in the crystallization condition. The sulfate ion likely competes with adenylosuccinate for binding at the *Lm*ASL active sites.

The catalytic C3 loop is disordered in our *Lm*ASL structure (Figure 2E), a state also observed in the unbound structures of the *H. sapiens* homolog (*Hs*ASL, PDB 4FFX and 4FLC [5], Figure S2). This region becomes ordered and structured upon ligand binding in the AMP and sulfate‐bound *Ld*ASL structure (AMP and sulfate‐bound PDB 4MX2 [8], figure 2E), which is also observed for the homologs from *E. coli* (AMP and fumarate‐bound, PDB 2PTQ [53]), *T. brucei* (AMP‐bound, PDB 4EFC [22]) and *H. sapiens* (AMP‐bound, PDB 4MX2 [8]; Figure S2). This structural transition suggests that the C3 loop functions as a dynamic gate that stabilizes the active site only during the catalytic cycle. Importantly, both the catalytic (His196, in the C2 region, and Ser321, in the C3 region) and the substrate‐binding amino acid residues (Arg40 and Tyr41 in the D1 domain, and Asn117, Asp119, Gln273, Asn335, Arg361, Ser366 and Arg370 in the D2 domain) are conserved in the *Lm*ASL amino acid sequence (Figure S1). A comparison of our *L. major* ASL structure (PDB 9BPL, this paper) with the AMP‐bound structure from *L. donovani* ASL (PDB 4MX2 [8]) revealed no major structural changes upon product binding (Figure 2F).

In both cases, domain D3 (Figure 2E) appears to be in an open position as defined for the unbound and the AMP‐bound *H. sapiens neanderthalis* ASL (PDB 5NX9 [51]). Importantly, Arg40 has been shown in *Ld*ASL to be critical for the activity and stability of the protein [8]. We confirmed that this residue is structurally conserved in *Lm*ASL (Figures 2F and S1). However, small differences were observed in the AMP binding site, with *Lm*ASL residues Asn117 and Asp119 away from the AMP binding position due to the flexibility of the loop where they are localized. Also, *Lm*ASL His196 sidechain is oriented away from the AMP site (Figure 2F).

We further observed that the adenylosuccinate/AMP and fumarate sites of the substrate/product‐bound *Hs*ASL crystal structure (PDB 5NX9 [51]) are conserved in the unbound *Lm*ASL (PDB 9BPL, this work) and the AMP‐bound *Ld*ASL (PDB 4MX2 [8]). We also observed two substitutions that may perturb the bonding network to the product: Asn335 and Asn117 in *Leishmania* ASL are replaced by larger positively charged amino acid residues Arg303 and Arg85, respectively, in *H. sapiens* ASL (Figure 2F). This finding could be used to design new compounds that selectively inhibit *Leishmania* ASL.

### 3.3 Allosteric Communications Within the *L. major* Adenylosuccinate Lyase Tetramer

To better understand the structure–function relationship of *L. major* *adenylosuccinate lyase*, we performed the network‐based analysis implemented in the Ohm webserver [29]. The Ohm algorithm [29] generated a perturbation propagation probability matrix based on atom contacts within the tetrameric structure of *Lm*ASL and determined allosteric coupling intensity (ACI) values for all residues. The OHM analysis successfully identified three distinct allosteric pathways connecting the catalytic site in monomer A to the N‐terminal regions of the other monomers. This connectivity suggests that the N‐terminal extension is not merely a structural stabilizer but a key component of the enzyme's inter‐subunit allosteric signaling.

**FIGURE 3:**
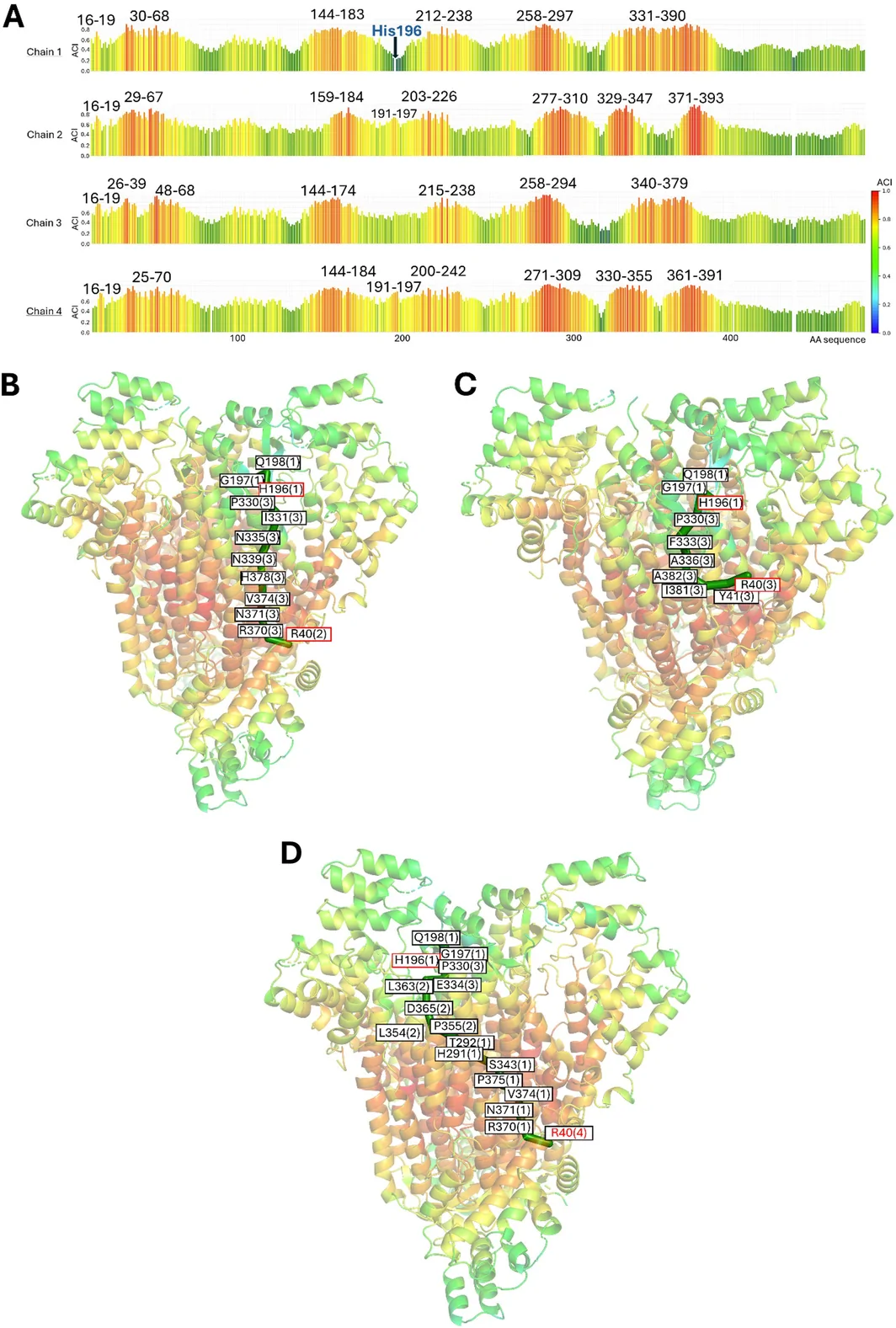
Allosteric communications within *Lm*ASL tetramer. (A) Bar charts showing allosteric coupling intensities (ACIs) calculated by Ohm [26] for each chain of the tetrameric structure of *Lm*ASL generated by PISA [24]. The active site (His196 from chain A) is depicted in blue. The color bar shows the ACI value range. (B–D) Critical amino acid residues in the most likely allosteric pathways (B‐ P1, C‐ P2 and C‐ P3) from each substrate binding/product site to the active site (set as His196 from chain A) are indicated. Protein structures are represented as cartoons, colored by ACI values.

Choosing His196 from the first chain of *Lm*ASL as the active site of interest, we observed prominent peaks in ACI values (colored red in Figure 3A) clustered around 6–7 allosteric hotspots along the amino acid sequence of each monomer (chains 1–4), mainly comprising the four substrate/product binding sites in the structure (Figure 3B–D). Moreover, comparable prominent peaks in ACI values were observed when symmetrically equivalent His196 residues were chosen as active sites of interest (Figure S3). These results indicate that the ligand interaction with each of the binding pockets is communicated throughout the protein to all the other three active sites. Through this computational approach, we sought to define the structural basis for cooperativity by identifying the network of residues that facilitate communication between distinct substrate‐binding pockets across the protein interfaces.

A similar analysis of the *Hs*ASL tetrameric structure (generated from PDB 4FFX [5], Figure S4) resulted in a comparable ACI distribution for all chains indicating a global allosteric mechanism for adenylosuccinate lyases. Interestingly, the ACI analysis in the N‐terminal region of *Lm*ASL shows prominent peaks around amino acid residues 25–70 (Figures 3A,B and S3). In fact, the essential amino acids residues Arg40 and Tyr41 from the first monomer are part of the AMP phosphate binding site (Figure 2F). Also, amino acid residues Asn335 and Arg370 from the third monomer, situated at the interface between two monomers, contribute to the interaction network at the AMP phosphate binding site (Figure 2F) and display high ACI values (Figure 3A). In contrast, Asp119 and Asn117 from the same monomer, which interact with the AMP ribose on the opposite side of the binding pocket, display lower ACI values. We mapped the amino acid residues that mainly contribute to the allosteric communication network within *Lm*ASL by independently selecting each one of the substrate/product binding pockets as putative allosteric sites (Figure 3B–D). The most likely allosteric communication paths are shown in Figure 3B–D. These allosteric paths share the amino acid residues Gly197 and Gln198 from the first chain, and Pro330 from the third chain, which are located in the upper part of the tetramer (Figure 3B–D). Importantly, path P1, extending from Arg40 (chain 2) to His196 (chain 1; Figure 3B), mostly comprises amino acid residues at the interface between chains 3 and 2. In contrast, path P2, spanning from Arg40 (chain 3) to His196 (chain 1; Figure 3C), is the shortest, involving only residues from chains 1 and 3. In contrast, path P3, extending from Arg40 (chain 4) to His196 (chain 1; Figure 3D), is the longest, incorporating residues from chains 1 through 4. Together, these results highlight the necessity of the enzyme's tetrameric form for its catalytic activity.

The allosteric communication network mapped by the ACI values aligns with the functionally critical regions of the ASL family. Specifically, the residues showing the highest communication pathways connect the active site His159 to the conserved C3 loop (residues 294–307) and the C1 region. These regions are known to undergo large‐scale conformational changes upon substrate binding across the aspartase/fumarase superfamily. The identification of these pathways suggests that *Lm*ASL utilizes this conserved allosteric ‘core’ to coordinate the structural transitions required for catalysis across the tetramer interfaces.

### 3.4 Kinetics Analysis of the *L. major* Adenylosuccinate Lyase

We further employed UV absorbance spectroscopy to evaluate the *Lm*ASL kinetics in vitro. Since other ASL enzymes do not follow Michaelis–Menten kinetics [5], we performed the Hill equation fitting to the enzyme kinetics data. The Hill plot resulted in a Hill coefficient of 1.1 ± 0.2. The kinetics parameters obtained were: *K*_M_ = 6.6 ± 0.6 μM, *v*_max_ = 2.22 ± 0.09 μmol.min^−1^, a *k*_cat_ = 48 ± 1 s^−1^, and a kinetic efficiency (*k*_cat_/*K*_M_) of (8 ± 1) × 10^6^ M^−1^ s^−1^ for the adenylosuccinate substrate at 25°C in a buffer containing 50 mM Tris–HCl at pH 8.5, consistent with the previously reported kinetics data for the *L. donovani* and *L. braziliensis* ASL [8, 49, 57] (Figure 1F). As expected, we did not detect any in vitro activity for SAICAR as a substrate.

### 3.5 Effect of the *Trypanosma brucei* ASL Knockdown in the Procyclic Form

**FIGURE 4:**
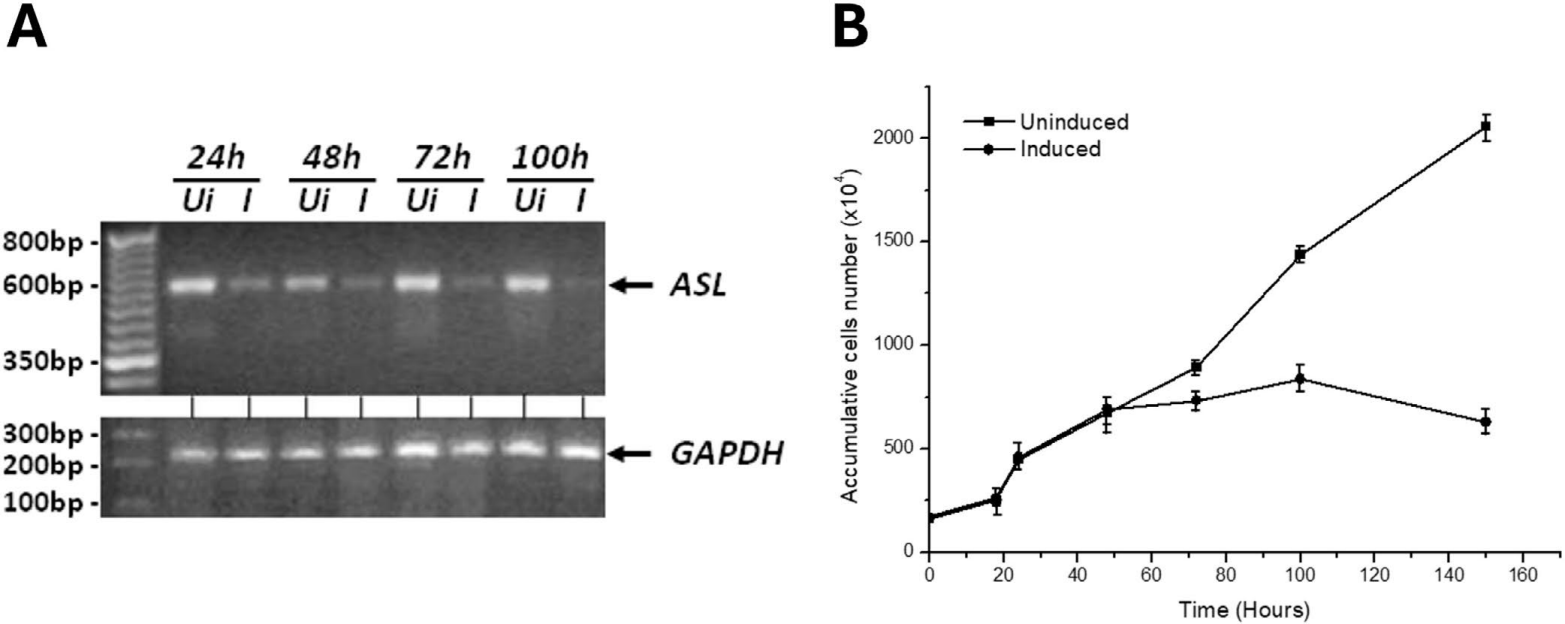
*Tb*ASL RNAi in procyclic *Trypanosoma brucei*. Effects of *ASL* knockdown on the growth rate of procyclic *T. brucei* 29–13 cells. (A) Reverse transcription‐PCR analysis of *ASL* mRNA levels after RNAi induction. The intracellular *ASL* mRNA levels were monitored from 24, 48, 74, and 100 h uninduced (Ui) and induced (I) samples, confirming that *ASL* mRNAs decreased, whereas levels of *GAPDH* control mRNA remained unchanged throughout the experiment. (B) Growth curves showing that 50 h after RNAi induction the cells interrupt their growth (●), while the cells from the uninduced culture continue to grow (■).

We examined whether *Tb*ASL is also essential for the procyclic *T. brucei* viability. We confirmed that the *Tb*ASL mRNA levels after RNAi induction were in fact much lower than the non‐induced control (Figure 4A), leading to a cell growth defect (Figure 4B), which agrees with the negative effect observed upon ASL knockout in *L. donovani* [57].

### 3.6 Identification of Potentially Druggable Regions in LmASL

Since trypanosomatid adenylosuccinate lyases are promising drug targets for trypanosomatiasis, we investigated the potential use of our crystal structure of *Lm*ASL for structure‐based drug design. We first performed an analysis of druggable sites in *Lm*ASL using the DoGSiteScorer server [58], identifying eight strong candidate pockets with druggability scores over 0.7 and a volume larger than 500 Å^3^. The identified sites comprise the substrate/product binding sites.

We selected the substrate binding pocket for molecular docking with the aim of finding small molecule competitive inhibitors of ASL that are selective to trypanosomatid ASL. Molecular docking against *Lm*ASL identified itraconazole (Figure S5, molecule 4), a phenylpiperazine‐derivative antifungal agent [59], one of the top‐performing ligands, with a GOLD docking score of 94.96 (rescore value of −90.82) against *Lm*ASL tetramer and the first lowest score (25.53) against *Hs*ASL tetramer (rescore value of −21.15), as we can see in the Table 2 with the 10 top‐performing ligands.

**TABLE 2:** The 10 top‐performing ligands by molecular docking score.

Molecule	*Lm*ASL score	*Lm*ASL rescore	*Hs*ASL score	*Hs*ASL rescore
1. Birinapant	94.96	−90.82	52.21	−55.16
2. N‐[(2,4‐difluorophenyl)methyl]‐N‐[2‐[(17‐methoxy‐5,7‐dioxa‐13‐azoniapentacyclo [11.8.0.02,10.04,8.015,20] henicosa‐1(21),2(10),3,8,13,15,17,19‐octaen‐16‐yl)oxy]ethyl]‐2‐(1,2,4‐triazol‐1‐yl)ethanamine	94.30	−79.50	57.21	−59.73
3. Isavuconazonium	91.62	−92.83	55.34	−62.87
4. Itraconazole	89.40	−91.51	25.53	−21.15
5. P‐113D	86.62	−82.68	−199.70	201.00
6. (2S)‐1‐[(2S,4R)‐4‐benzyl‐2‐hydroxy‐5‐[[(1S,2R)‐2‐hydroxy‐2,3‐dihydro‐1H‐inden‐1‐yl]amino]‐5‐oxopentyl]‐N‐tert‐butyl‐4‐(furo[2,3‐b]pyridin‐5‐ylmethyl)piperazine‐2‐carboxamide	84.85	−79.30	63.86	−63.65
7. Q203	83.83	−81.97	27.86	−28.75
8. 3‐[2‐(4‐aminophenyl)ethyl]‐8‐benzyl‐7‐[2‐[ethyl(2‐hydroxyethyl)amino]ethyl]‐1‐propylpurine‐2,6‐dione	82.96	−83.83	56.09	−48.04
9. Saquinavir	82.03	−74.84	34.18	−33.93
10. Ketoconazole	81.72	−75.89	64.73	−71.70

**FIGURE 5:**
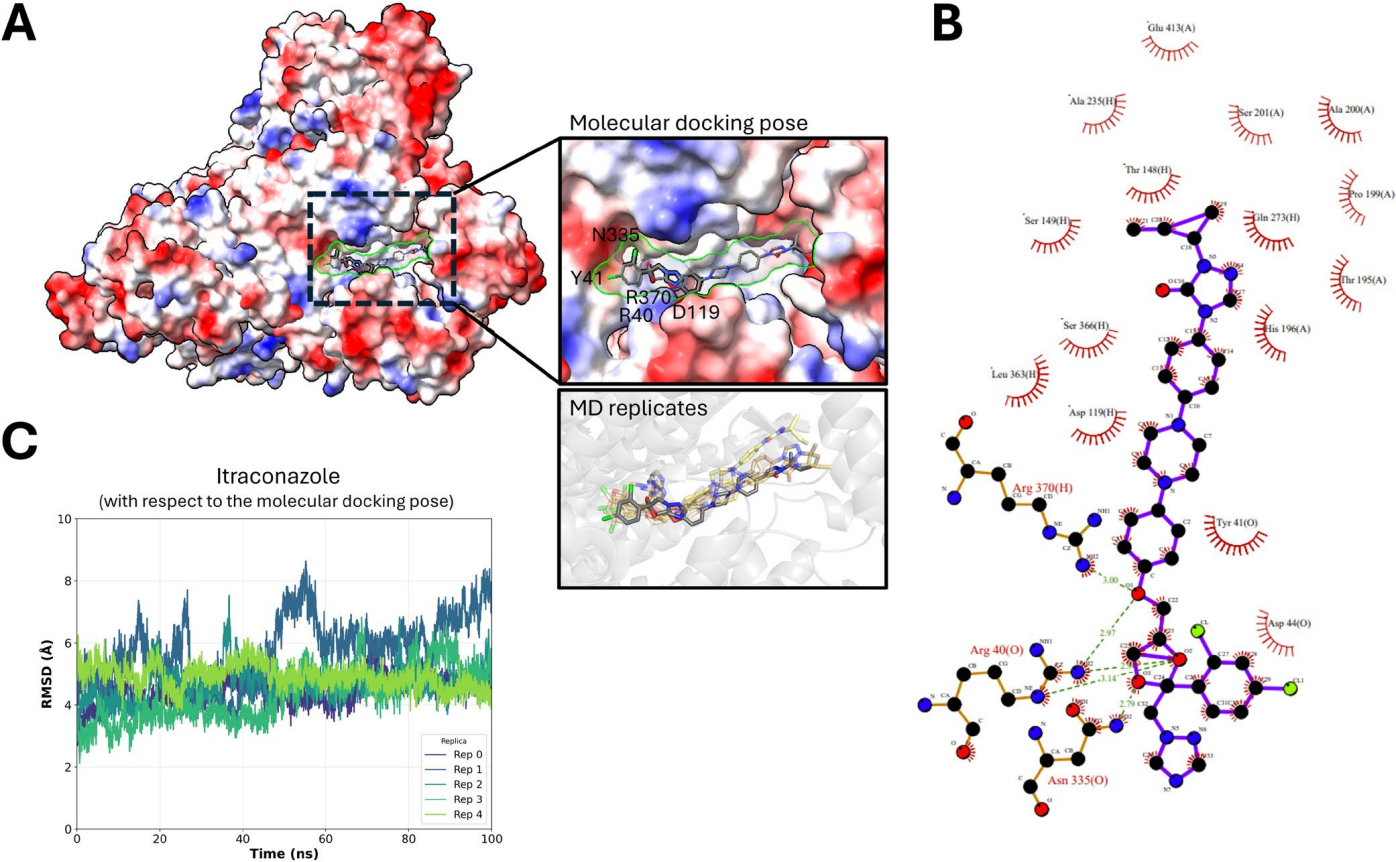
Itraconazole binding mode to *Lm*ASL. (A) Electrostatic surface potential representation of *Lm*ASL (obtained using APBS [25]) bound to itraconazole shown as sticks (highlighted in green). Molecular dynamics (MD) simulation poses of five independent replicates are also shown as sticks. Structural analyzes were performed in Chimera [42] and PyMOL Molecular Graphics System (Version 3.0, Schrödinger LLC). (B) LIGPLOT of interactions involving itraconazole, generated by PDBSum [60]. (C) Root‐mean‐square deviation (RMSD) of the itraconazole atomic positions during the MD simulations of five independent replicates.

To reinforce the predicted mode of binding, a second round of molecular docking using a different software, CBDock [35], was performed using both flexible and rigid ligand structures against *HsASL* (26% sequence identity with *Lm*ASL) and *Lm*ASL. For both proteins, the new poses were very similar to the reference one obtained from the first round of docking (RMSD_*Lm*ASL_ = 0.71 Å, RMSD_*Hs*ASL_ = 0.56 Å). Despite the good agreement of the poses, the distinct scores of 33.55 (rescore value of −23.17) and 65.93 (rescore of −67.38), respectively, for *Hs*ASL and *Lm*ASL reinforce a possible selectivity to the *L. major* enzyme, an essential feature for the development of new treatments with low side effects. The polar regions of itraconazole anchor it to the substrate binding pocket of *Lm*ASL, mainly through contacts of the dichlorophenyl group with amino acid residues Arg40 and Tyr41, and the piperazine group with Asp119 (Figure 5A,B).

To assess the reliability of the predicted binding pose of the itraconazole molecule to *Lm*ASL obtained by molecular docking, we conducted five independent 100 ns molecular dynamics (MD) simulations. The ligand root‐mean‐square deviation (RMSD) appeared relatively high during the trajectory (average RMSD: 4.8 Å, minimum average RMSD = 4.3 Å, maximum average RMSD = 5.7 Å; Figure 5C). This deviation primarily reflected an early ligand reorientation within the binding pocket (Figure 5A) rather than unbinding or instability (Figure S6A). Specifically, the methyl‐propyl triazolone moiety exhibited a consistent shift from its initial interaction with residue Ala235 toward residue Lys432. The initial distance measured in the docked pose (using the *Lm*ASL unbound crystal structure as a template) was 7 Å. During the molecular dynamics (MD) simulations, this distance increased to an average of 14.7 Å during the simulation and then stabilized around 6 Å (Figure S6B) in four out of five replicates. This convergence occurred rapidly and was maintained throughout the simulation, suggesting a more favorable binding configuration for this moiety. This ligand transition appears to be facilitated by a conformational rearrangement of the Lys315‐Pro325 loop, a flexible segment absent in the crystallographic structure of the unbound protein. Despite exhibiting diverse conformational sampling across the simulations, as revealed by principal component analysis (PCA; Figure S7), the loop adopted a convergent bent conformation in two replicates. In this state, the loop bends toward the α‐helix spanning residues Gly437‐Lys432 (initial inter‐loop distance: 19.9 Å; final average distance: 14.98 Å; maximum average distance 17.3 Å; minimum average distance: 12.9 Å; Figure S6B), resulting in a reshaped binding site that more effectively accommodates the ligand.

In contrast, the opposite region of the ligand demonstrated considerable stability throughout the simulations. The dioxolane ring maintained a consistent interaction with residue Thr367, as reflected by a low variance in interatomic distance (initial distance: 5.1 Å; final average distance: 5.23 Å; minimum average distance: 4.62 Å, maximum average distance: 5.7 Å; Figure S6C). Similarly, the dichlorophenyl group remained closely associated with residue Arg43 over time (initial distance: 10.3 Å; final average distance: 6.7 Å; minimum average distance: 5.5 Å; maximum average distance: 8.4 Å; Figure S6D).

Itraconazole scores 1 on Lipinski's rule of five, with a molecular weight of 705.6 Da, although it can still be considered a small molecule. Its molecular structure contains several resonance points, 14 highly electronegative atoms (Cl, O, N), and five aromatic rings (Figures 5B and S5, molecule 4). *Lm*ASL shares only 26% sequence identity with *Hs*ASL, which likely contributes to the lower ligand binding affinity for *Hs*ASL, making it more effective at targeting preferentially the parasite protein. Although itraconazole selectively binds to the substrate binding pocket of *Lm*ASL, as observed in the molecular docking experiments, further experiments are necessary to validate this small molecule as a bona fide ligand of the enzyme. Nonetheless, it can be used as an initial hit in the rational development of competitive inhibitors of the trypanosomatid ASL function.

Additionally, our model of the homotetramer complex of *Lm*ASL presents an additional promising target region for the rational drug design: small molecules could be designed to selectively interact with the cleft on the monomer surface where the adjacent N‐terminal region extension binds. Together, the differences between amino acid residues observed in the *Leishmania* ASL and the *H. sapiens* homolog indicate a potential candidate for in silico HTS strategies to develop inhibitors against *Leishmania* ASL using a structure‐based approach.

## 4 Discussion

In this paper, we focus on the adenylosuccinate lyase (ASL), an enzyme of the purine synthesis pathway of the parasitic protozoa *Leishmania major*. The purine synthesis pathway is necessary for the parasite to utilize host purines, thus meeting its purine needs. ASL is essential for AMP production, especially under conditions where hypoxanthine, inosine, guanine, and guanosine are the major purine sources [7]. Therefore, ASL is crucial for the survival and growth of *Leishmania* parasites under controlled conditions and in infected mice [10] and thus is highly regarded as a potential drug target. Although other enzymes of the purine synthesis pathway such as transferases, including hypoxanthine‐guanine phosphoribosyl transferase (HGPRT), could theoretically be exploited in the selective drug design against *Leishmania*, their essentiality has not been demonstrated, and therefore they are not currently considered validated drug targets [57]. In this study, we further determined that ASL is essential for procyclic *T. brucei* growth in the presence of hypoxanthine in the growth medium.

The phylogenetic reconstruction of ASL proteins, by Maximum Likelihood, highlights the evolutionary distance between the Kinetoplastida and the host ASL, reinforcing the possibility of the parasite ASL being a feasible drug target. Our structural and biochemical analysis confirms that *Lm*ASL is active as a homotetramer as observed for other homologs, and its structure is highly similar to its bacterial counterparts and less similar to its human homolog. The parasite‐specific N‐terminal extension contributes to a robust inter‐subunit interface, potentially enhancing tetramer stability compared to the human homolog, and may offer a unique site for selective allosteric interference. An analysis of the allosteric communication network within the unbound *Lm*ASL in comparison to the unbound *Hs*ASL revealed that all four substrate/product binding sites are interconnected and communicate with each other. This result explains the need for the formation of a tetramer for the enzymatic function. Furthermore, we revealed the presence of an unprecedented N‐terminal extension binding to a cleft in each adjacent monomer to improve monomer‐monomer interactions in the homotetramer.

Differences in the substrate/product binding site amino acid residues could be used in structure‐based drug design approaches to identify specific and selective inhibitors of *Lm*ASL. To test this hypothesis, we performed an *in silico* molecular docking screening campaign using a library of 400 ligands from the Pandemic Response Box (PRB) [36]. We identified a phenylpiperazine derivative molecule, itraconazole, as a candidate for the selective inhibition of the *Leishmania* ASL compared to human homolog. This molecule is anchored to the substrate binding site through residues that include the specific Arg40, which has been shown as essential for *L. donovani* ASL function [8]. While our structural analysis and MD simulations suggest that the unique N‐terminal extension of LmASL could serve as a docking site for small molecules like itraconazole, we acknowledge that its role as a functional inhibition site remains a structural hypothesis. Further experimental validation, such as the characterization of N‐terminal deletion mutants or site‐directed mutagenesis of the interface, is required to definitively establish the functional relevance of this region in enzymatic regulation. Further studies are necessary to validate this small molecule as a potential hit that could be developed into a drug candidate for leishmaniasis. Furthermore, new structure‐based drug design approaches may target the N‐terminal extension binding to adjacent ASL monomers, which we propose to be specific for trypanosomatid ASL.

Studies on adenylosuccinate lyase inhibitors are scarce, and most focus on the human enzyme. One of the earliest examples is the classic work by Bridger and Cohen [61], who investigated the effect of the 6‐mercaptopurine nucleotide (thioinosinate or T‐IMP) as a competitive inhibitor of *Hs*ASL. Inhibition occurs due to its structural similarity with the enzyme's natural substrate, but the molecule has low specificity and affects multiple purine pathways, limiting its potential as a selective and therapeutic inhibitor. Furthermore, this compound was only studied in human models, without considering the structural differences between human and parasite ASL enzymes, such as that from *Leishmania major*.

More recently, Crifò et al. [62] described that *Hs*ASL, especially in the presence of mutations associated with enzyme deficiency, can be modulated by reactive aldehydes like trans‐4‐hydroxy‐2‐nonenal (HNE), which result from oxidative stress. However, this type of modulation represents a nonspecific interaction, unrelated to rational enzyme inhibition or therapeutic applications in parasitic infections.

The structure‐based approach presented here represents a more targeted starting point for the development of specific antiparasitic drugs, overcoming the limitations of specificity and applicability observed in previous studies. Although further validation is needed, this structure‐based approach contributes a distinct angle to ongoing drug discovery efforts against leishmaniasis by incorporating enzyme selectivity into the earliest steps of compound screening. This may help bridge existing gaps between classical ASL inhibitors and the need for parasite‐specific therapeutics.

Together, our data shows new insights into the structure–function relationship of *Leishmani* ASL, indicating druggable sites that will be targeted in future studies focused on designing potent inhibitors of adenylosuccinate lyase function in the purine synthesis pathway of *Leishmania* spp. In addition, we also showed that procyclic *T. brucei* depends on ASL for its survival, which corroborates the negative effect on *Leishmania* growth upon ASL knockout [10].

## Author Contributions

**Ivan R. e Silva:** methodology, investigation, writing – original draft, formal analysis, data curation. **Monique Mantovani:** investigation, methodology, formal analysis, data curation. **Maria E. S. D. Lino:** investigation, methodology, formal analysis, software, data curation. **Aline O. Albuquerque:** investigation, methodology, software, formal analysis, data curation. **João H. M. da Silva:** investigation, methodology, software, formal analysis, data curation. **Ronaldo A. P. Nagem:** investigation, methodology, software, formal analysis, data curation. **Adriana L. Rojas:** investigation, methodology, formal analysis, data curation. **Geraldo R. Sartori:** investigation, methodology, formal analysis, data curation. **Eduardo Horjales:** investigation, supervision. **David Campbell:** investigation, supervision. **Otavio H. Thiemann:** conceptualization, funding acquisition, writing – original draft, project administration, supervision, formal analysis, validation.

## Funding

This work was supported by Fundação de Amparo à Pesquisa do Estado de São Paulo, 2018/20693‐4, 2013/17791‐0; Conselho Nacional de Desenvolvimento Científico e Tecnológico, 403598/2021‐4.

## Disclosure

Accession Code: The atomic coordinates and structure factors of *Lm*ASL have been deposited in the Protein Data Bank [26] with accession code 9BPL.

## Conflicts of Interest

The authors declare no conflicts of interest.

## Supporting information


**Table S1:** Root mean square deviation (RMSD) and amino acid sequence identity of *Lm*ASL and its orthologs.
**Figure S1:** Multiple sequence alignment of selected ASL amino acid sequences with a corresponding crystal structure deposited in PDB.
**Figure S2:** Comparative analysis of the C3 loop region conformation in the absence and in the presence of substrate.
**Figure S3:** Allosteric communications within the unbound LmASL tetramer.
**Figure S4:** Allosteric communications within the unbound HsASL tetramer.
**Figure S5:** Structure of the top 10 scoring ligands in molecular docking experiments.
**Figure S6:** Analysis of molecular dynamics (MD) simulations.
**Figure S7:** Principal Component Analysis (PCA).

## Data Availability

All data are available from the authors upon request. Please send request to Ivan Rosa e Silva ivan.silva@alumni.usp.br, Otavio H. Thiemann thiemann@ifsc.usp.br.
