# Supplementary material for: Structural Insights Into the Function of Leishmania major Adenylosuccinate Lyase
Source: Proteins. 2026 Jun 8;94(10):1686–701. doi: 10.1002/prot.70150 (PMC13539675; doi:10.1002/prot.70150)
Supplement: Supplementary file 1 [file PROT-94-1686-s001.docx]

**SUPPLEMENTARY INFORMATION**

**Structural Insights into the Function of *Leishmania major* Adenylosuccinate Lyase**

Ivan R. e Silva^a,b*^, Monique Mantovani^a,c^, Maria E. S. D. Lino^a^, Aline O. Albuquerque^d^, João H. M. da Silva^d,e^, Ronaldo A. P. Nagem^f^, Adriana L. R. Cardona^a,g^, Geraldo R. Sartori^a^, Eduardo Horjales^a^, David Campbell^h^, Otavio H. Thiemann^a*^

^a^ Institute of Physics of São Carlos, University of São Paulo, São Carlos, São Paulo, Brazil

^b^ Present address: Brazilian Biosciences National Laboratory (LNBio) at the Brazilian Center for Research in Energy and Materials (CNPEM), Brazil

^c^ Present address: Libbs Farmacêutica Ltda, Brazil

^d^ Laboratory of Structural and Functional Biology Applied to Biopharmaceuticals, Fundação Oswaldo Cruz, Fiocruz Ceará, Brazil

^e^ Pasteur-Fiocruz Center on Immunology and Immunotherapy, Fundação Oswaldo Cruz, Fiocruz Ceará, Brazil

^f^ Department of Biochemistry and Immunology, Federal University of Minas Gerais, Belo Horizonte, Minas Gerais, Brazil

^g^ Present address: CIC bioGUNE, Bizkaia Science and Technology Park, building 800, Derio (Bizkaia)

^h^ Microbiology, Immunology & Molecular Genetics, University of California, Los Angeles, USA

* Corresponding authors: Ivan Rosa e Silva [ivan.silva@alumni.usp.br](mailto:ivan.silva@alumni.usp.br), Otavio H. Thiemann, [thiemann@ifsc.usp.br](mailto:thiemann@ifsc.usp.br).

**Table S1 – Root mean square deviation (RMSD) and amino acid sequence identity of *Lm*ASL and its orthologs.**

| **Organism** | **PDB ID** | **RMSD (Å)** | **Sequence identity (%)** |
| --- | --- | --- | --- |
| *Escherichia coli* | 2PTS | 0.93 | 50 |
| *Plasmodium vivax* | 2HVG | 0.89 | 43 |
| *Salmonella typhimurium* | 4NSL | 0.82 | 50 |
| *Legionella pneumophila* | 3BHG | 0.70 | 47 |
| *Trypanosoma brucei* | 4EFC | 0.66 | 70 |
| *Leishnmania donovani* | 4MX2 | 0.51 | 96 |

**Supplementary Figure Legends**

**Figure S1. Multiple sequence alignment of selected ASL amino acid sequences with a corresponding crystal structure deposited in PDB.** Regions C1-C3 and domains D1-D3 are indicated. The arrows point to substrate/product binding amino acid residues. Asterisks point to active site amino acid residues. Amino acid residues are colored by conservation score. Figure adapted from ENDScript (<https://endscript.ibcp.fr/>; Robert, X. and Gouet, P. (2014). DOI: 10.1093/nar/gku316) analysis using default parameters.

**Figure S2. Comparative analysis of the C3 loop region conformation in the absence and in the presence of substrate.** Cartoon representation of the ASL active site depicting the C3 loop conformation. Unbound ASL: black: *Hs*ASL (PDB 4FFX [5]) and grey *Hs*ASL (PDB 4FFC [5]). AMP-bound ASL: blue: *Ec*ASL (PDB 2PTQ [52]), magenta: *Ld*ASL (PDB 4MX2 [8],), orange: *Tb*ASL (PDB 4EFC [22]) and black: *Hs*ASL (PDB 5NX9 [50]).

**Figure S3. Allosteric communications within the unbound *Lm*ASL tetramer.** Cartoon representation of the tetrameric structure of *Lm*ASL colored by allosteric coupling intensities (ACIs) calculated by Ohm [26]. The active site (His196 from chains 2, 3 and 4, respectively) is indicated. ACI values are displayed from low (0, blue) to high (1, red) values.

**Figure S4. Allosteric communications within the unbound *Hs*ASL tetramer. A-** Bar charts showing allosteric coupling intensities (ACIs) calculated by Ohm [26] for each chain of the tetrameric structure of *Hs*ASL generated by PISA [24] from the structure with PDB accession number 4FFX ([5]). Chain 1: A; Chain 2: B; Chain 3: C; Chain 4: D. The selected active site was the amino acid residue His159 from chain A. The color bar shows the ACI value range. **B**- Cartoon representation of the tetrameric structure of *Lm*ASL colored by ACI values.

**Figure S5. Structure of the top 10 scoring ligands in molecular docking experiments.**

**Figure S6. Analysis of molecular dynamics (MD) simulations. A-** Root-mean-square deviation (RMSD) of the mainchain atomic positions of the itraconazole-bound *Lm*ASL protein over time during the five independent replicates of MD simulations. Temporal analysis of the distances between **B**-residue Ala235 and atom C19 of the itraconazole, **C-** Thr367 and atom C26 of the ligand, and **D-** the centroid of the carbon atoms from 2,4-dichlorophenyl moiety and the guanidinum group of Arg43, shown in stick + ribbon representations of the protein-ligand complex.

**Figure S7. Principal Component Analysis (PCA).** PCA of the loop positions spanning from amino acid residues Asp311-Pro330 over five molecular dynamics simulations replicates of the *Lm*ASL-itraconazole complex. Itraconazole molecules are shown as sticks and the interacting loops are presented as ribbon as insert. Green and blue circles denote the initial and final frames of the simulation, respectively, while red X marks indicate the most frequently sampled conformation within each replicate.

**Figure S1**

**Figure S2**

**Figure S3**

**Figure S4**

**Figure S5**

**Figure S6**

**Figure S7**
